# “Our lab is the community”: Defining essential supporting infrastructure in engagement research

**DOI:** 10.1017/cts.2018.325

**Published:** 2018-11-27

**Authors:** Donald E. Nease, Dee Burton, Sarah L. Cutrona, Lauren Edmundson, Alex H. Krist, Michael Barton Laws, Montelle Tamez

**Affiliations:** 1 Department of Family Medicine, School of Medicine, Colorado Clinical and Translational Sciences Institute, University of Colorado – Anschutz Medical Campus, Aurora, CO, USA; 2 Center for Health, Media and Policy, Hunter College, City University of New York, New York, NY, USA; 3 Department of Quantitative Health Sciences, Division of Health Informatics and Implementation Science, University of Massachusetts Medical School, Worcester, MA, USA; 4 Center for Healthcare Organization and Implementation Research, Edith Nourse Rogers Memorial Veterans Hospital, Bedford, MA, USA; 5 Harvard T.H. Chan School of Public Health, Cambridge, MA, USA; 6 Department of Family Medicine and Population Health, Virginia Commonwealth University, Richmond, VA, USA; 7 School of Public Health, Brown University, Providence, RI, USA; 8 Colorado Clinical and Translational Sciences Institute, University of Colorado, Aurora, CO, USA

**Keywords:** Patient-centered outcomes research, community-engaged research, community-based participatory research, research infrastructure, stakeholder-engaged research, community engagement

## Abstract

**Introduction:**

Effective patient engagement is central to patient-centered outcomes research. A well-designed infrastructure supports and facilitates patient engagement, enabling study development and implementation. We sought to understand infrastructure needs from recipients of Patient-Centered Outcomes Research Institute (PCORI) pilot grant awards.

**Methods:**

We surveyed recipients of PCORI pilot project awards on self-perceived strengths in engagement infrastructure through PCORI’s Ways of Engaging-Engagement Activity Tool survey, and interviewed leaders of 8 projects who volunteered as exemplars. Descriptive statistics summarized the survey findings. We conducted a thematic analysis of the interview transcripts.

**Results:**

Of the 50 surveyed pilots, 22 answered the engagement infrastructure questions (44% response rate). Survey and interview findings emphasized the importance of committed institutional leadership, ongoing relationships with stakeholder organizations, and infrastructure funding through Clinical and Translational Science Awards, PCORI, and institutional discretionary funds.

**Conclusions:**

These findings highlight the importance of and how to improve upon existing institutional infrastructure.

## Introduction

Although there is a long tradition and considerable literature concerning community-based participatory research (CBPR) in community-level health research and intervention studies [1–4], the practice of stakeholder engagement in clinical research, including patient-centered outcomes research, is a more recent innovation. It is not clear how concepts derived from the CBPR tradition translate to this setting. Stakeholder engagement is a key component of patient-centered outcomes research. It requires long-term collaboration with patients and families, care teams, and community members, yet there is a lack of systematic information describing the necessary infrastructure to sustain these relationships.

The benefits of stakeholder engagement in health research have drawn increased attention in recent years through the Agency for Healthcare Research and Quality’s Effective Health Care Program (effectivehealthcare.ahrq.gov), the National Center for the Advancement of Clinical and Translational Science’s Clinical and Translational Science Award program’s central focus of “engaging communities in defining healthcare needs and in receiving the benefits of research” [5, 6], and the centrality of stakeholder engagement to the mission of the Patient-Centered Outcomes Research Institute (PCORI). The Veterans Administration has also undertaken an initiative to engage Veterans in the development, implementation, and dissemination of research studies [7]. The growth in this area of research is shown in the increasing number of publications. A PubMed search in November 2017 on the keywords “community engagement” and “translational research” yielded 235 citations, nearly all of them published after 2000, and the majority after 2010.

As we use it here, the term “infrastructure” encompasses not only the “bricks and mortar” infrastructure covered through indirect cost recovery funds but also resources, policies, relationships, and culture within (and extending beyond) the research organization. For example, to obtain research funding, the specific aims and research strategy must be fully developed with a high level of scientific rigor prior to proposal submission. Ideally, patients and other stakeholders at this stage are engaged in the proposal development; however, involvement at this state is challenging without financial support and other resources [8]. Similarly, training and experience is important for all participants to be able to work together effectively [9].

There is a lack of systematic information about stakeholder engagement infrastructure. Much has been written about the challenges and facilitators of engagement and about engagement best practices; while infrastructure is often mentioned in this work, the empirical basis for this writing is limited [3, 10, 11]. Studies also describe evaluations of engagement, a process that requires an understanding of the success of many infrastructure elements [12]. Further work is needed, however, to understand the existing infrastructure for stakeholder engagement, to describe how academic and community partners use and experience that infrastructure, and to identify those infrastructure components that are most valuable.

PCORI made its initial awards in 2012, under the rubric of “pilot projects.” Many pilot awardees were leaders in the field of engagement research, and given their experience were well-positioned to offer insights into critical components of engagement infrastructure. Our research sought to identify and describe research engagement infrastructure elements that were present and that potentially contributed to the success of PCORI pilot awardees. With our findings, we seek to equip organizational leaders with information that will facilitate institutional support for future stakeholder engagement efforts and successful engagement research.

## Methods

Our project began with discussions at a meeting for the PCORI pilot project learning network in March 2014, where all pilot project awardees gathered to collaborate and share lessons from their work. The authors recognized that there was insufficient information in the literature addressing the infrastructure needed to support engagement research. With the help of AcademyHealth staff, we began a series of conference calls to define our methods.

We defined “engagement infrastructure” as “the resources, policies, relationships and culture at a research organization that facilitate better, easier and more effective engagement of patients and community members for the purpose of informing the design, conduct and dissemination of Patient-Centered Outcomes Research.” After reviewing the literature, we next defined the domains of research engagement infrastructure, as shown in Table 1.

**Table 1:** Domains of research engagement infrastructure

Domain	Examples
Skills and expertise in engagement methods	Investigator and staff with engagement training and expertise Ongoing use of demonstrated engagement methods Development of novel engagement methods
Training for researchers and patients/community members	Training programs and workshops Community immersion training IRB 101 for patients and community members
Connections and relationships	Relationships developed formally or informally with community partners that are continuous and transcend individual projects
Staff to support engagement activities	Community outreach staff
Longitudinal, nonstudy-dependent engagement activities	Joint academy/community advisory board
Culture that supports engagement	Commitment at multiple levels of the organization to principles of engagement Supportive leadership
Engagement policies and procedures	Supportive IRB policies Defined roles, responsibilities, and engagement policies Evaluation policies Data collection and sharing policies
Systems promoting effective dissemination of study findings in conjunction with community partners	Advisory board meetings and presentations Media contacts Communications staff Community Research Forum (annual gala event with posters from community projects) Include community members as co-presenters at academic meetings Web site/blog
Funding/resources	CTSA support Financial support for engaged patients Institutional support

CTSA, Clinical and Translational Sciences Award; IRB, Institutional Review Board.

We used a mixed-methods approach, combining quantitative (traditional Likert survey) data with qualitative (free text survey and key informant interview) data for the next phases of our study. First, we surveyed PCORI pilot awardees regarding the presence or absence of strengths in each infrastructure domain within their local organization. PCORI surveyed pilot project awardees from January to March 2015, using the Ways of Engaging-ENgagement ACtivity Tool (WE-ENACT). We were given the opportunity to add additional items at the end of the survey pertaining to our study. We asked respondents to rate the “quality of each component of engagement infrastructure at their institution” based on our research engagement infrastructure domains. We provided response options on a 5-point Likert scale ranging from 1=poor to 5=excellent. Respondents were also asked to explain their ratings using a free text box for each item, in order to gather more qualitative data in support of the survey responses. Finally, each respondent was given the opportunity to self-identify as an exemplar willing to participate in a subsequent key informant interview about the infrastructure at his or her organization.

Next, we conducted in-depth interviews with the self-identified exemplars from the WE-ENACT survey. A semi-structured interview guide was developed for a ~30-min interview with exemplars. Interviews were conducted by 2 members of the study team (D.E.N. and M.B.L.) during the summer of 2015. Interviews were transcribed and stored securely as text and audio files.

Interviews were analyzed using immersion and crystallization methods with thematic coding being applied to specific passages of text. Specifically, 2 members of the study team performed purposive coding of each interview assigning codes to granular quotes. Following this, another 2 members of the study team reviewed those codes, going back to the original interview transcripts as well, and developed a refined set of themes. Investigator M.B.L. then conducted a second analysis exploring these themes using the qualitative data analysis software Atlas.ti™ (Scientific Software Development GmbH) to elucidate how the themes are realized in the institutions of the specific respondents.

## Results

Among the 50 pilot award principal investigators, 27 completed the PCORI WE-ENACT survey and 22 answered the engagement infrastructure questions (44% response rate). Table 2 displays the mean ratings of infrastructure quality by infrastructure domain along with the summaries of the free text comments written by respondents from each domain. Respondents rated the quality of “connections and relationships” domain highest and “funding/resources” lowest at their institutions. No domain was rated at or above “good” on average, and standard deviations were relatively wide, reflecting a broad distribution of ratings for each domain.

**Table 2:** Ways of Engaging-Engagement Activity Tool respondent mean ratings of the “quality” of local engagement infrastructure with summarized comments (n=22)

Domain	Mean (SD)	Summarized survey comments
Connections and relationships	3.68 (1.25)	Most respondents stated that they have good connections with patients and other stakeholders. They cited their own programs (e.g., community-engaged research core), stakeholder groups, and individual stakeholders as the means through which they make connections. One respondent noted that being embedded in a health delivery system makes this process easier. Two respondents noted a lack of connections, one because they are a new organization and the other because they focus on methods research
Skills and expertise in engagement methods	3.58 (1.26)	Respondents cited several types of infrastructure that support their skills and expertise in engagement methods, such as a Center for Disease Control prevention research center, a Patient-centered Comparative Effectiveness Research Center, and a Clinical and Translational Science Award (CTSA) community-engaged research core. Several noted their experience in participatory research. Two respondents emphasized the quality and experience of their research team, although their institutions as a whole may not be as strong
Culture that supports engagement	3.37 (1.03)	Most explanations of culture that supports engagement highlighted opportunities for improvement. Several respondents suggested formalizing the culture. Some noted that their own team is supportive, but others in the institution are less supportive. One respondent was positive about their culture, simply stating that they have a “fun and engaged team”
Longitudinal, nonstudy-dependent engagement activities	3.32 (1.43)	Respondents pointed to programs that support ongoing engagement activities, including an action board, a community-engaged research core, and involvement in another Patient-Centered Outcomes Research Institute (PCORI)-funded project
Systems promoting effective dissemination of study findings in conjunction with community partners	2.89 (1.26)	Respondents pointed to different types of resources that support dissemination, such as their public relations department or communications staff, a speakers’ bureau of patient advocates, and collaboration with national patient advocacy organizations. One respondent stated there is room for improvement at their institution
Engagement policies and procedures	2.79 (1.12)	Most responses about policies and procedures were positive. One respondent noted that their Institutional Review Board (IRB) is “very receptive” to creating new policies around engaged research, and other said their policies are “helpful and supportive.” Only one respondent stated that they do not have such policies in place
Training for researchers, patients, and community members	2.68 (1.26)	Many respondents emphasized a lack of progress in this area. Some institutions have IRB training for investigators but noted a dearth of such programs for stakeholders. A few stated that they have no such program at all. One respondent’s institution is home to many training and grant opportunities, and others identified their community-engaged research core and Patient-Centered Comparative Effectiveness Research Center as good training resources
Staff to support engagement activities	2.68 (1.27)	Respondents noted several ways in which staff support their engagement activities. Two teams mentioned programs at their institutions, one for family partners in research and a community-engaged research core. Another group stated that their partner agency provided key support in their research. One respondent noted that their staff are helpful but do not adequately “infiltrate” the institution
Funding/resources	2.58 (1.16)	Most respondents emphasized inadequate funding. They noted that institutional funding is decreasing or does not exist at all. One respondent indicated that investigators cannot depend on such support. Another emphasized that PCORI’s reporting requirements were challenging for financial staff to work with. One project noted that their support comes from their community-engaged research core

*Five-point Likert scale ratings ranging from 1=poor to 5=excellent

Eight individuals agreed to be interviewed as exemplars, all of whom were principal investigators for their projects, with the exception of one individual who was a master’s level study coordinator. Themes that emerged from the interview analysis confirmed the identified engagement domains. In addition, “leadership” emerged as a strong theme within the domain of “culture.” Table 3 displays the domains, exemplar themes, and key quotations that we identified.

**Table 3:** Exemplar interview quotes, cited infrastructure elements, and factors in relationship to domains (n=8)

Domain	Infrastructure elements and factors that facilitate engagement
Connections and relationships	*Quote: “Go out and talk to people. That’s how you’ve got to start with building relationships”*
	Longstanding academic/state partnerships Existing patient/community advisory councils Existing relationships with stakeholders and community partners Existing practice-based research networks Existing programs focused on community engagement and partnerships
Skills and expertise in engagement methods	*Quote: “…frankly, some people aren’t cut out for this type of work, which is very important to recognize”*
	History of engagement work Existing programs focused on community engagement and partnerships Pockets of engagement expertise within institution staff with experience Existing relationships with qualitative researchers Understanding that engagement in care is different than engagement in research
Culture that supports engagement	*Quote: “…and the position we take, which is very counterculture, if you will, is that the question shouldn’t be how do you engage the community in your research, but how do you as researchers get yourself engaged in what the community is doing”*
	Culture values relationships with patients and communities above all else Institutional culture has supported engagement research for a long time Have organizational support for engaging patients Culture of working collaboratively with patients Organization is interested in patient perspective Culture focused on patients having the answers to understanding disease
Longitudinal, nonstudy-dependent engagement activities	*Quote: “…the biggest single thing that I learned from our advisory council members…was that they were not in it for the money or recognition…what was essential to all of them was that what they said was taken seriously and actually made a difference”*
	Center that existed for many years Longstanding infrastructure for research collaboration
Systems promoting effective dissemination of study findings in conjunction with community partners	*Quote: “It really does take a village to be effective in engagement”*
Engagement policies and procedures	Best practices are based on experience, but not codified Published “standards” of patient centeredness IRB protocol that could be used for community partnerships Partnering with fiscal agent facilitates getting money to community partners Consumer dominated board of directors
Training for researchers, patients, and community members	
Staff to support engagement activities	Existing personnel resources beneficial Named engagement officer at hospital
Funding/resources	*Quote: “You have to have the scanners and the chromatography equipment even if there’s no particular study yet; you just need that investment to get the infrastructure up to maintain your lab. Well, our lab is in the community”*
	CTSA helpful for pulling together community-engaged work, providing resources and infrastructure and impacting institutional culture AHRQ funding for practice-based research networks NIH-funded P30 core center Success with PCORI funding Have resources from the university State Health Department has given some funds CTSA offers grants to help build relationships with communities Grant focused on community relationships CTSA supporting grants that fund both investigators and community partners Able to get seed funding for projects, such as $25,000 Grants support pre-project work Dean provided bridge funding to continue a working relationship with community residents

AHRQ, Agency for Healthcare Research and Quality; CTSA, Clinical and Translational Science Award; IRB, Institutional Review Board; NIH, National Institutes of Health; PCORI, Patient-Centered Outcomes Research Institute.

The respondents varied at the institutional level which they represented, and these varied perspectives led to different levels of focus for their descriptions of engagement infrastructure within their institutions. In some cases, respondents described the infrastructure of individual research programs; in other cases, respondents focused on departments or an entire school. An illustrative response from respondent 4 was:

*Obviously, we have the Department of Family Medicine which has its research program. Nested within that is the stuff we do through our PBRN [Practice-Based Research Network]. And then I direct a center which is separate from the Department of Family Medicine that … does a lot of community-engaged research. So when you ask a question like how… dominant in our culture is… patient engagement or other stakeholder engagement I sort of have different answers depending on what lens I’m looking through.*

Respondent 2 felt that the stakeholder engagement was well entrenched in community health research at his institution, but not in clinical research. “I can see little pockets in the institution that has some expertise. But in my experience and my PCORI grant I was really on my own.” Respondent 1 reported that she was associated with a center for research on a specific disorder which has strong ongoing relationships with the affected community and a commitment to stakeholder engagement, but she could not speak to other parts of her university. Similarly, respondent 3, who was with a center for research on a specific disease and was associated with an international organization, reported that patient engagement was now institutionalized in research in his center:

*So the group, when it was founded … included clinicians, included representatives from the pharmaceutical industry … included regulators from Europe and the United States … but a decade into …the group – this is about 12 years now, a number of people in leadership said, “Well, we probably should go to patients … And based on the patient involvement at that point, it - the patients said, “Well, you’re not including anything to do with fatigue or with sleep or with these other things that are actually important to us as we live with the disease… . And from that point forward, patients are actually included as an essential component of the meeting, and actually in terms of the meeting itself, patients constitute about 10% of the total representatives at any given biannual meeting.*

Respondent 5 was associated with a research and education institute within a large, vertically integrated healthcare system. He saw stakeholder-engaged research as an emerging priority within his organization:

*I should also have added is that we were started by a concerned citizen organization in 1956. We’re not just nonprofit, but we’re one of the few such organizations, health systems, that has pretty much a patient, excuse me, a consumer-dominated Board of Directors.*

Respondent 6 was affiliated with a medical school which has an established center for community partnership that works to generate CBPR. She felt that individual researchers’ relationships with community organizations were even more “powerful.” The school also had a Clinical and Translational Sciences Award (CTSA), which included a community engagement core. Respondent 7 was in a Department of Family Medicine which worked with practice-based research networks that had patient advisory councils; the school also had a CTSA with a community engagement core. Respondent 8 was the director of the CTSA community engagement core at her university. In that role, she worked to promote stakeholder-engaged research, but the CTSA is no longer funded and she did not believe that stakeholder-engaged research had become well established at the institution.

Many respondents indicated that leadership was critical to establishing an institutional culture that supports stakeholder-engaged research. As respondent 3 said:

*I do think that we are patient centered in our overall philosophy and approach and that comes from the highest level of our division. You know…it also comes from the…dean of our campus, he always says that medicine is a public trust. And, you know, that in medicine, your obligation is back to the patient and that should be the center of everything you do.*

However, respondent 3 also said that incentives for faculty were not well aligned with stakeholder engagement. “Publishing things regarding engagement is not easy… The academic rewards, which is judged in grants and publications, is not being matched by the ability to get stuff out there.” Other respondents also pointed to this obstacle. Respondent 4 emphasized the importance of leadership but suggested that leaders may need persuading:

*For organizations that are starting out it’s also important that they deal with the matter of culture that you’ve identified in this survey. I think that’s extremely important getting support from leadership if you’re at an academic or other institution. I think it’s crucial if you are going to succeed in pulling in those resources or leveraging resources that already exist at the institution. So there needs to be a strategy for how to make the case and establish that culture that is supportive of that kind of work.*

Several respondents also pointed to the ongoing relationships with community organizations and representatives. As respondent 4 put it:

*The position we take, which is very countercultural,…is: the question shouldn’t be “how do you engage the community in your research” but “how do you as researchers get involved in what the community is doing?” How do you get yourself at the table where everybody else is bringing their various skills to bear on how to improve population health in the community…? So, it clashes with the traditional culture on our campus and I’m sure on many others and at NIH.*

With the exception of respondent 8, interviewees generally believed that support for engagement research was increasing. As respondent 6 stated, “You know, really partnering with patients and stakeholders, I would say, is just much more part of the norm than it ever was.” Respondent 7 said, “Some people aren’t cut out for this kind of work [but] those who are cut out for it are working very hard to make it the norm. And I think we’re being successful that way.” Several mentioned PCORI as a driver of increased interest and activity in engagement research.

These comments highlight a need for permanent resources to support stakeholder engagement. As respondent 4 said, “You have to have the scanners and the chromatography equipment even if there’s no particular study yet; you just need that investment to get the infrastructure up to maintain your lab. Well, our lab is in the community.” Respondents found various resources for sustaining work with stakeholders. The CTSA for respondent 2 has a planning grant mechanism, which is split between researchers and community partners, and has also received support from the dean’s discretionary funds. Echoing respondent 4, respondent 2 said, “This is mostly the kind of funding that will pay for a microscope…but this patient panel…we built it as a microscope.” Respondent 3 has a patient-centered outcome hub as part of a National Institutes of Health funded P30 core. Respondent 7’s practice-based research networks were dependent on individual project grants for funding (after initial support from the agency for healthcare research and quality), but were able to maintain a continuous flow. Respondent 7’s institution also had a CTSA, which supported engagement. Many respondents also mentioned PCORI as an important funder.

## Discussion

Our research sought to define the essential research engagement infrastructure using the cohort of investigators funded by PCORI’s pilot awards as a study population. Using a set of domains defined through a review of the literature, our participants most strongly identified the connections and relationships, the skills and expertise in engagement methods, and a culture that supports engagement as being of higher quality at their organizations. These survey responses were supported by our interviewees, who most strongly highlighted the importance of leadership, which we identified as an element of a culture that supports engagement. Funding to support the ongoing relationships with stakeholders and to develop projects emerged as also being very important in our interviews. Finally, most interviewees voiced optimism about the overall trajectory of research engagement and the infrastructure available to support the work.

The themes of connections and relationships, skills and expertise, and a culture that is supported by active leadership can be seen as being intertwined and being sustained by ongoing funding. Skills and expertise in engagement research are required to successfully build and maintain longitudinal connections and relationships with stakeholders. Funding that ebbs between grant awards makes it difficult to sustain longitudinal relationships with stakeholders. Thus, a key element of leadership support of a culture that fosters engagement research may include core institutional funding. Absence of a supportive culture fostered by leaders at various organizational levels, funding and policies to support research engagement may limit even early efforts and success.

Our interviewees touched on almost all of our identified engagement themes, with the exception of training. It may be that training was perceived by our interviewees as being necessary to achieve skills and expertise in research engagement. Still, it was not specifically mentioned. Although training was in the lower third of ratings for our infrastructure domains, it is important to note that neither training nor any of our lower rated domains were rated as having a “poor” level of quality at respondents’ organizations.

This research has limitations in that we studied a sample of investigators who had received PCORI pilot awards. This was the first cohort of PCORI awardees, and may not be fully representative of all investigators conducting engagement research. Our response rate of 44% was also somewhat low, possibly due to the length of the survey. Both of these factors may limit the generalizability of our research. More work is needed to confirm our findings in other groups of investigators.

In contrast, the use of mixed methods which combined quantitative survey items with free text survey responses and subsequent key informant interviews with self-identified exemplars is a strength of our study. As noted, the interviews, in particular, supported the survey findings, while expanding upon these with important detail from interviewees experiences.

Respondents clearly were working in institutions with a broad spectrum of infrastructure and history related to engagement research, and their responses are reflective of that diverse context. However, their responses provide consistent evidence of the importance of research engagement infrastructure, especially in the domains of culture and leadership, skills and expertise to conduct the research and ongoing connections and relationships with stakeholders. These elements require support of ongoing, secure funding. Institutional and organizational leaders who wish to build and foster the growth and maintenance of research engagement should take note of our findings.
